# Improvement of Bond Strength and Durability of Recycled Aggregate Concrete Incorporating High Volume Blast Furnace Slag

**DOI:** 10.3390/ma14133708

**Published:** 2021-07-02

**Authors:** Shu-Ken Lin, Chung-Hao Wu

**Affiliations:** 1 Department of Civil Engineering, National Chung Hsing University, No. 145 Xingda Rd., South District, Taichung City 40224, Taiwan; sklin@nchu.edu.tw; 2 Department of Civil Engineering, Chung Yuan Christian University, No. 200, Zhongbei Rd., Zhongli District, Taoyuan City 32023, Taiwan

**Keywords:** recycled aggregated concrete, blast furnace slag, bond strength, interfacial transition zone, water permeability, chloride-ion penetration, durability

## Abstract

This paper aims to experimentally investigate the effects of high volume cement replacement of blast furnace slag (BFS) on the bond, strength and durability of recycled aggregate concrete (RAC). Concrete mixtures were prepared containing 0%, 15%, 30%, 45%, 60% and 75% BFS with each of recycled aggregate and natural aggregate. Measurements of the compressive and bond strength, the resistance to chloride-ion penetration and the water permeability of concrete are reported. In addition, a microhardness test was also performed to evaluate the quality of interfacial transition zone (ITZ) in concrete. Test results of the bond strength and the compressive strength of RAC mixtures, in spite of the cement replacement amount with BFS, show that the concretes result in reduced strength when compared to natural aggregate concrete (NAC) mixtures, while the strength gains for the BFS-based concrete are higher than that of the reference mixtures without BFS at long-term ages. Incorporating BFS in concrete can inherently improve the durability properties by increasing higher resistance to chloride-ion penetration and lower water permeability. This improvement in the mechanical and durability properties of the BFS-based RAC mixture may be due to the additional pozzolanic reaction of BFS, which enhances the properties of ITZ in concrete, resulting in an improvement of the strength of concrete.

## 1. Introduction

Cement and aggregate are the two main components in concrete. However, the procedures of their production emit much CO_2_ and consume a large amount of energy, which is unfriendly to the natural environment. In the past decades, many researchers intended to find an effective method for producing sustainable concrete with a low amount of cement or recycled aggregate. The general measure is to crush waste concrete as coarse aggregate for producing concrete, which was regarded as recycled aggregate concrete (RAC) [1,2,3]. The surface of recycled coarse aggregate is generally full of pores, presenting high water absorption, which affects the interfacial transition zone (ITZ) property in concrete, leading to negative effects for the durability of concrete [4,5,6,7]. In order to improve the durability of the RAC, pozzolans such as fly ash and blast furnace slag were added to concrete; nevertheless, there are restrictions on the dosages of the pozzolans in many national standard codes [8,9,10].

For past decades, increasingly more research results of concrete with high volumes blast furnace slag (BFS) were reported, with most discussing natural aggregate concrete [11,12,13,14,15], while few dealt with RAC incorporating high replacement levels of BFS, especially on the durability aspect. Djelloul [16] investigated the improvement of workability of self-compacting concrete (SCC) mixes containing recycled aggregates with increasing BFS from 0 to 30%. They found that recycled coarse aggregate (RCA) content of 25% to 50% natural aggregate replacement and cement replacement of 15% BFS may be the best dosage to produce available SCC without any segregation or bleeding. El-Hawary [17] reported the results of experimental research on the durability and performance of various mixtures of RAC incorporating 25% BFS as cement replacement. The test results reported that incorporating mineral slag improved the mechanical properties and durability characteristics of the RACs. Addition of 25% BFS evaluated to be efficient in reducing water absorption, alkali–silica reactivity and sulfate attack in RAC. However, the experimental results also presented that introduction of 25% BFS is not effective enough to prohibit the excessive expansion of the alkali–carbonate reaction in RAC after one year. Furthermore, it has an adverse impact on resisting the drying shrinkage and water permeability of the RAC mixtures.

Hence, some studies further investigated the effect of high volume blast furnace slag. Khodair [18] examined the effect of recycled coarse aggregate (RCA) on the properties of SCC. Twenty RAC mixtures with different replacement ratio of RCA, BFS and fly ash (FA) were prepared and tested. The test results showed that the replacement ratio of recycled aggregate increased, the compressive strengths of concrete decreased at 3, 14, and 28 days. Moreover, the partial replacement of cement by pozzolans presented an adverse effect on the 28-day RAC compressive strength; however, it increased the resistance to chloride permeability.

Majhi [19] researched the effects of RCA and BFS on fresh and hardened concrete properties; 16 concrete mixtures were prepared with 0–100% replacement of natural aggregate by RCA for each 0–60% replacement of cement by BFS. The test results showed that the workability decreased with the use of RCA. The compressive, flexural and split tensile strength decreased with the increase in the replacement ratio of RCA or BFS or both. Voids and water absorption in the concrete mixtures increased with the increase of RCA content. However, the use of BFS enhanced the quality of the concrete by improving the interfacial transition zone and the bond strength between mortar and aggregate. The concrete mixture with 50% RCA and 40% BFS achieved properties similar to those of the natural aggregate concrete without BFS.

In addition, the bond strength of recycled aggregate concrete is also an interesting issue. Some studies discussed the bond strength between RAC and rebar in comparison with that of ordinary concrete [20,21]. Their test results indicated that the reduction of bond strength could be associated with the increment of recycled aggregate used in the mixture, which presented reductions of 6–8% up to 30% of bond strength. In order to improve the bond strength of RAC, some researchers added pozzolan into concrete to enhance the bond strength. Majhi [22] investigated concrete mixes containing 0%, 40% and 60% BFS with each of 50% and 100% RCA. The test results showed that the compressive strength of RAC can satisfy the 28-day compressive strength requirements of the concrete grades of M15, M20 and M25, as per IS 10262 (2009) [23]. Majhi [24] also investigated the mechanical properties and durability of RAC utilizing high volume BFS (up to 60% replacement ratio of cement) with lime activator. The results revealed that the enhancement in the mechanical properties of blast furnace slag-based RAC mixes were found to be in the range of 14.02–19.61%, 10.74–14.71%, 9.33–14.07% and 6.65–14.17% for compressive, flexural and bond, and splitting tensile strength, respectively.

Furthermore, other studies focused on discussing the microstructure at steel–concrete interface in reinforced concrete. Soylev [25] studied the influence of steel–concrete interface defects on reinforcing steel corrosion. Their test results showed that the defects related to the gaps caused by segregation, bleeding and settlement of fresh concrete under horizontal reinforcing bars (RB). These defects increased with the concrete depth below the horizontal RB and depended on the bleeding capacity of the concrete mixture. Horne [26] reported that compared with the bulk cement paste, the aggregate–cement-paste and RB–cement-paste interfaces presented more porosity and calcium hydroxide (CH) and less unreacted cement. As the hydration age increased, the porosity near the interfaces decreased, and the CH increased with more CH close to the RB than to the aggregate. Chen [27] indicated that there was a relatively wide porous band with large voids and pores near the RB–concrete interface. They also found that size of porous band around the RB is not uniform, and that the water to cement ratio can significantly affect the distribution and size of porous band at the interface.

The abovementioned research suggests that RAC with high replacement levels of BFS may present improved durability and mechanical properties at later age. This study aims to further investigate the fresh properties, mechanical properties and durability of RAC that incorporates high replacement levels of BFS. The mechanical properties of hardened concrete were evaluated by testing the interfacial transition zone performance at the surface of aggregate and steel bars, which were measured using microhardness tests [28,29].

## 2. Materials and Methods

### 2.1. Materials

(1) Cement: Type I Portland cement obtained by Taiwan Cement Corporation Ltd., Taipei, Taiwan, with a specific gravity of 3.15. The basic properties of cement are shown in Table 1.
(2) Blast furnace slag (BFS): Grade 120 of ground granulated blast furnace slag obtained from Advanced-Tek Systems Co., Ltd., Taipei, Taiwan. The basic properties of BFS are shown in Table 1.
(3) Fine Aggregate (FA): Natural river sand with a specific gravity of 2.60 and fineness modulus of 2.40.
(4) Coarse aggregate (CA): Crushed river stone with a specific gravity of 2.61, bulk density of 1470 kg/m^3^ and maximum size of 19 mm.
(5) Recycled coarse aggregate (RCA): Crushed waste concrete with a specific gravity of 2.26, bulk density of 1280 kg/m^3^ and particle sizes of 5~20 mm.
(6) Superplasticizer (SP): High performance water-reducing agent obtained from HI CON Chemical Admixture Taiwan Ltd., Taipei, which meets the CNS 12283 Type G with a specific gravity of 1.2 ± 0.02 and pH 7.0 ± 1.0.

### 2.2. Mixture Proportion and Specimen Preparation

The mixture proportions of concrete were designed according to ACI 211.1 [30], basically, for providing the compressive strength of natural aggregate concrete (NAC) of 41 MPa at 28 days. The water to cementitious material ratio (w/cm) ranged from 0.30 to 0.50, and the BFS content ranged from 0% to 75% by weight of the total cementitious materials as cement replacement. The proportions of a dozen kinds of mixtures are shown in Table 2.

**Figure 1:**
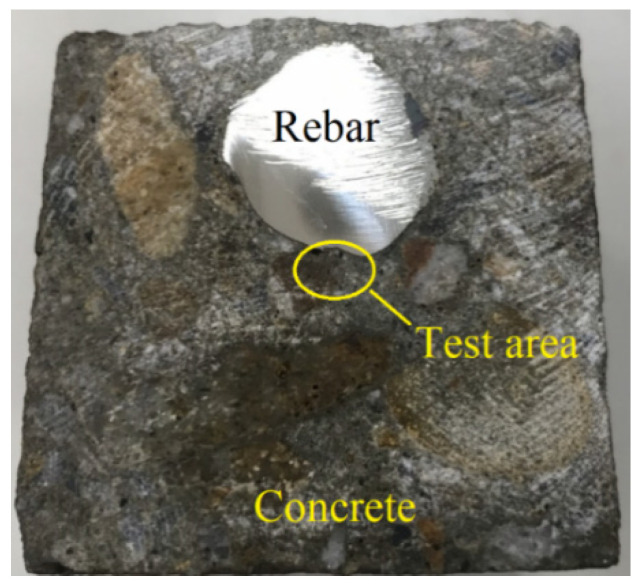
Vickers hardness test section of cube specimen.

The fresh properties of concrete, including slump, air content and unit weight, were simultaneously measured for each batch. Specimens for testing were cast from each mixture: a cylinder specimen of φ100 mm × 200 mm for compressive strength test, cylinder specimen of φ150 mm × 50 mm for water permeability test and cylinder specimen of φ100 mm × 50 mm for chloride-ion permeability test. For the bond strength test, cubes of 150 mm size were prepared by embedding a reinforcing bar of #7 (nominal diameter 22.2 mm) vertically along the central axis of the specimen. A cube specimen of 50 mm × 50 mm × 50 mm in dimension with a #4 (nominal diameter 12.7 mm) bar placed horizontally in the middle was prepared for the microhardness test. Figure 1 shows the cut section of specimen for testing. After removal from the molds, all specimens were moved to a standard moist-curing room until date for testing.

### 2.3. Test Procedures

The compressive strength test was performed according to ASTM C 39 [31] at the ages of 7, 28, 56 and 91 days. Steel pull-out testing (ASTM C234 [32]) for bond strength determination was carried out at 7, 28, 56 and 91 days. The resistance of concrete to the penetration of chloride ion was measured using rapid chloride-ion permeability test (RCPT, ASTM C1202-12 [33]), in terms of the charge passed through the concrete in coulombs. The water permeability of concrete (IS 3085 [34]) was tested using a water permeability apparatus subjected to a water pressure of 0.29 MPa for 3 h to determine the flow through the specimen. The chloride-ion penetration and water permeability tests were carried out at 28, 56 and 91 days. Vickers hardness test was performed according to ASTM E384-17 [35] for recognizing the existence and properties of ITZ. The Vickers microhardness HV (MPa) is calculated as:

(1) $$\mathrm{HV}=\frac{P}{\mathrm{As}}=2P\frac{\sin(\frac{\alpha }{2})}{d^{2}}=\frac{1.8544P}{d^{2}}$$

where As = surface area of indentation (mm^2^), P = load (N), *α* = face of angle of indenter at 136° and *d* = mean diagonal of indentation (mm). Generally, the hardness is correlated with the strength of material tested.

## 3. Experimental Results and Discussion

### 3.1. Fresh Properties of Concrete

The measured fresh properties of concrete including slump, air content and unit weight are shown in Table 3. It shows that the concrete mixtures were mixed with superplasticizer to produce a workable slump in the range of 210 to 250 mm. The air content of concrete ranged from 1.2% to 3.4% for natural aggregate concretes and 1.8% to 4.8% for RACs, respectively, basically increased with the increase of the cement replacement ratio of BFS. The unit weight of concrete ranged from 2215 kg/m^3^ to 2314 kg/m^3^ decreased as the replacement ratio of BFS increased. These results indicate that the RAC mixtures prepared for test can exhibit adequate properties of fresh concrete.

### 3.2. Mechanical Properties of Concrete

#### 3.2.1. Compressive Strength

**Figure 2:**
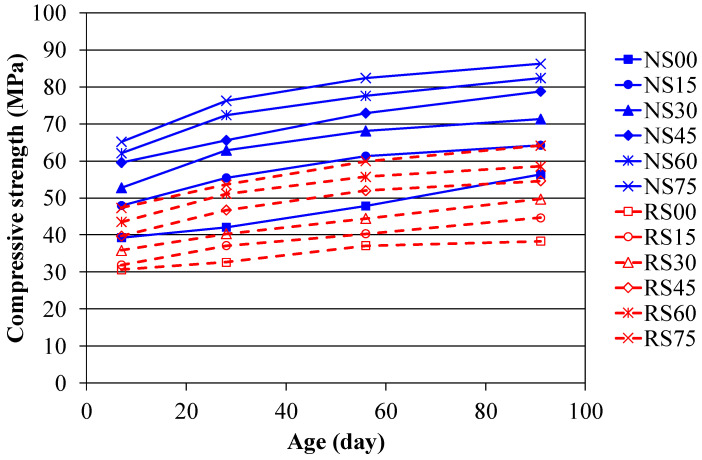
Compressive strength development of concrete.

The compressive strength of concretes measured at various ages is shown in Table 4; Figure 2 shows the development of compressive strength for each concrete mixture. It is seen that the strength development with age of the NAC and RAC mixtures present a similar trend, in which the compressive strength values of RAC series were all lower than that of the corresponding mixtures of NAC series. This indicates that despite the cement replacing amount with BFS, the mixtures of RAC result in reduced strength compared to that of the NAC.

It can be seen in Figure 2 that the curve slope of the BFS mixtures tends to be steeper than that of the reference mixture without BFS at later age. In other words, the strength gain for the BFS mixtures is higher than that of the reference mixture without BFS. This improvement in the long-term compressive strength may be the consequence of the pozzolanic reaction of BFS, leading to enhance the strength.

#### 3.2.2. Properties of ITZ in Concrete

Microhardness testing has been reported to be a means for characterizing the bulk paste properties in cement pastes, especially for the properties of interfacial transition zone (ITZ). The ITZ was recognized as a significant part of the microstructural system in concrete, which plays an important role in affecting the properties of concrete, such as compressive strength, tensile strength, fracture and permeability.

**Figure 3:**
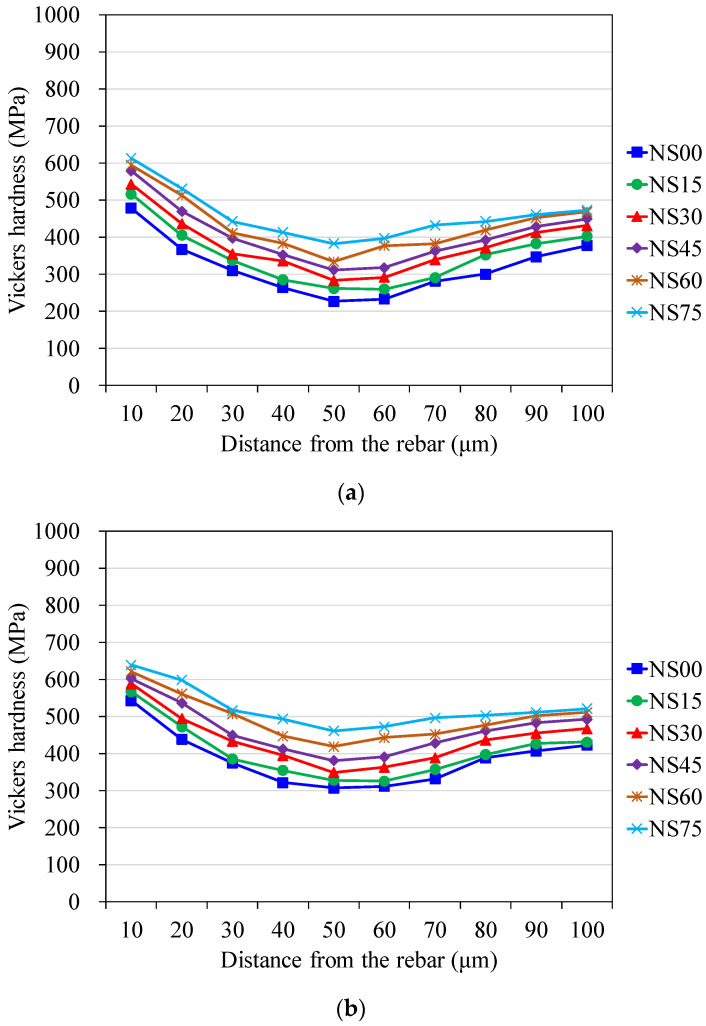

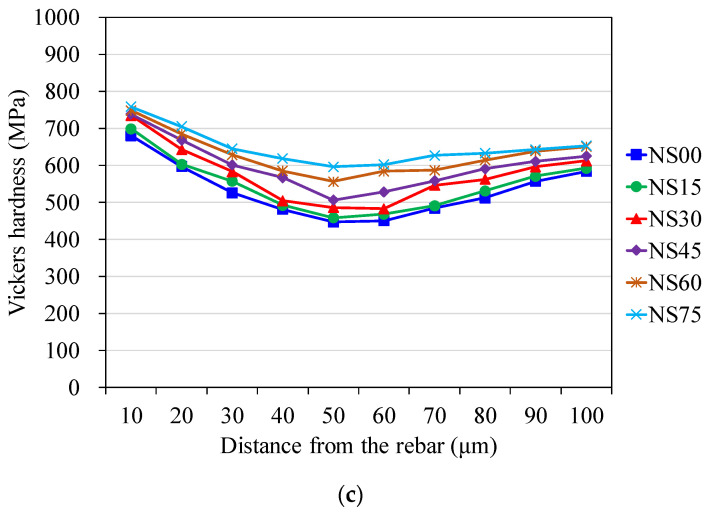
Measured Vickers hardness of normal aggregate concrete at ages of (**a**) 28 days, (**b**) 56 days and (**c**) 91 days.

**Figure 4:**
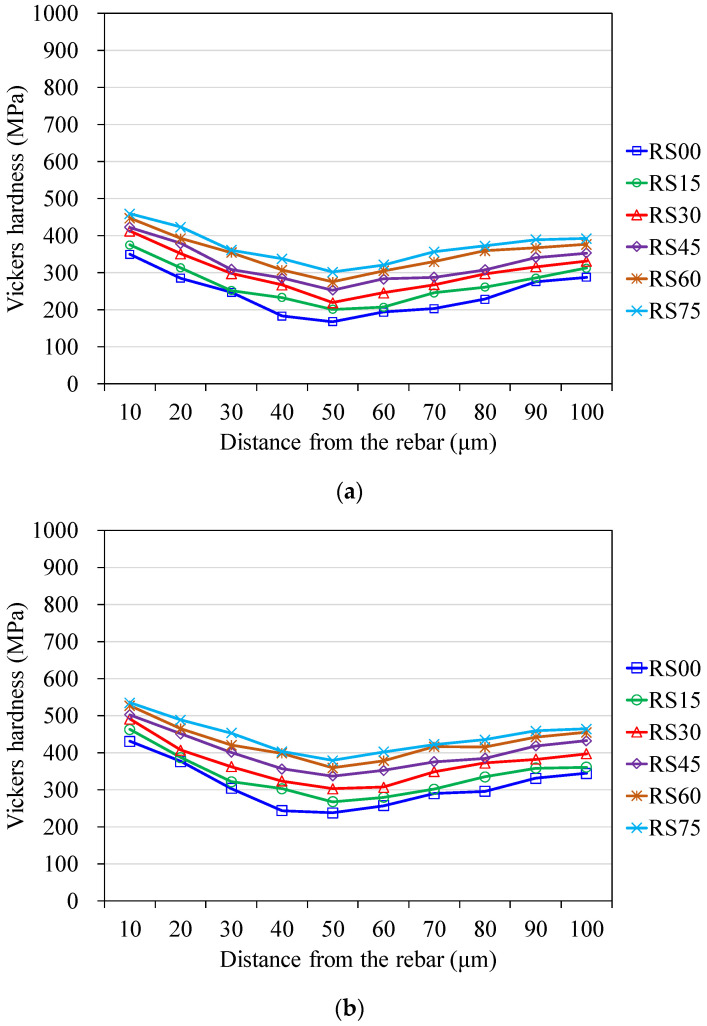

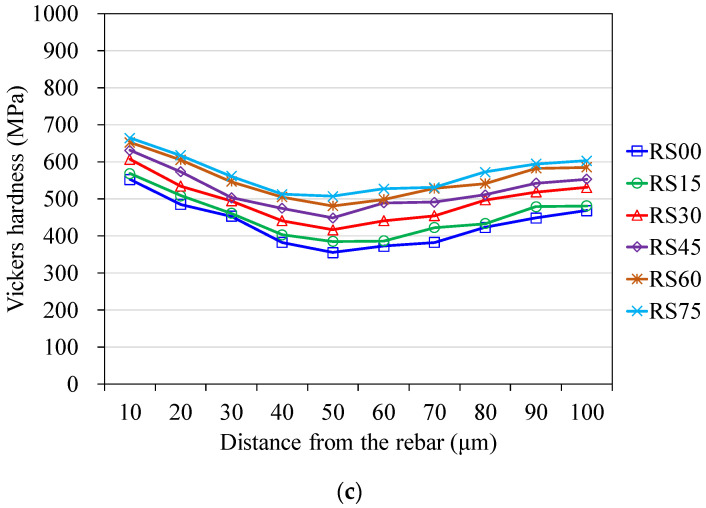
Measured Vickers hardness of recycled aggregate concrete at ages of (**a**) 28 days, (**b**) 56 days and (**c**) 91 days.

Figure 3 and Figure 4, respectively, illustrate the microhardness profiles of measured results for NACs and RACs. All curves show that in the vicinity of the rebar surface there is a gradient in microhardness, but in the outer bulk paste it tends to be constant. The width of the zone, with a varied gradient, may be referred to as ITZ. Figure 3 and Figure 4 show that both NAC and RAC mixtures at all ages present a similar curve shape of ITZ. The distinction from each other of any two ITZ curves displays solely in the form of depression; deeper depression represents a lower microhardness value. Both RAC and NAC mixtures incorporated with various BFS content show higher microhardness at ITZ than that of the correlated control mixture without BFS. Furthermore, for each age, concrete containing more BFS presents higher microhardness at ITZ; the concrete mixture with 75% BFS achieves the highest microhardness. These results indicate that incorporating BFS makes the ITZ in concrete denser and stronger, especially at long-term ages. This may infer to be due to the latent hydraulic activity and the additional pozzolanic reaction of BFS, which enhance the properties of ITZ and in turn the strength of BFS concrete.

#### 3.2.3. Bond Strength

The bond strength of concrete obtained from the pull-out test was calculated as:

(2) $$\mu_{avg}=\frac{P}{\pi \cdot d_{b}\cdot L_{e}}$$

where *μ_avg_* is the average bond stress (MPa), P is the measured maximum load (N), *d_b_* is the diameter of rebar (mm) and *L_e_* is the length of embedment (mm).

Table 5 summarizes the results of the pull-out test measured at the ages of 7, 28, 56 and 91 days. It is seen that the average bond stress *μ_avg_* (also named bond strength) of concrete increased with the increase of compressive strength. The bond strength of BFS concretes was higher than that of the reference concrete without BFS, which increases with the increase in BFS-based content. This increase in bond strength of BFS concrete may be due to the denser and stronger ITZ at the surface of steel bars, as discussed earlier.

**Figure 5:**
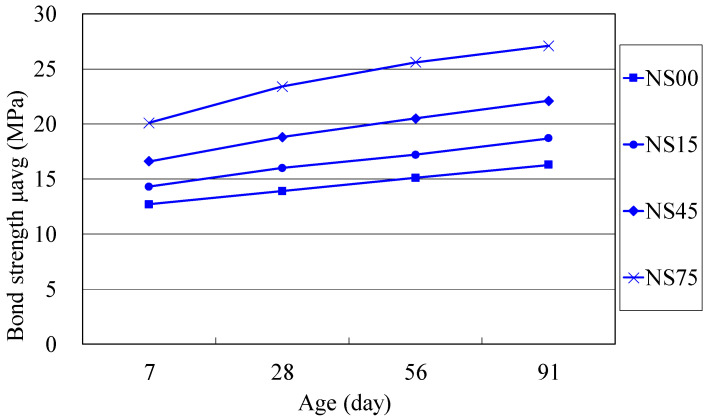
Bond strength of NAC versus curing age.

**Figure 6:**
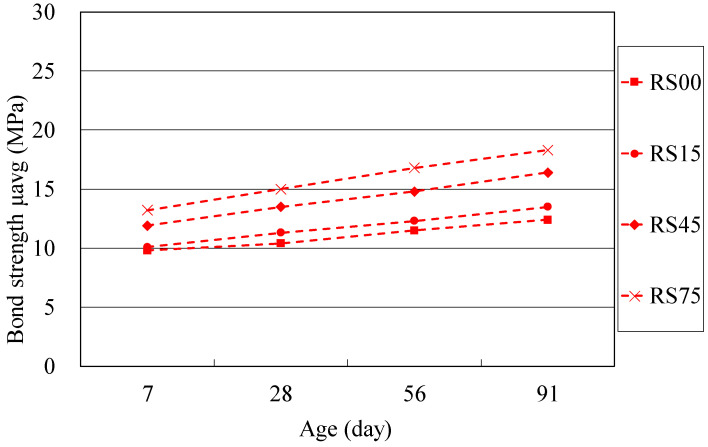
Bond strength of RAC versus curing age.

To evaluate the long-term effect of BFS on the bond strength of concrete, the bond strength gains (in percentage) of each mixture series at 28, 56 and 91 days with respect to the 7-day bond strength are also calculated, as shown in Table 5. Their bond strength development with age of NAC and RAC mixtures are illustrated in Figure 5 and Figure 6. From Table 5, it is found that the rate of bond strength gains of each BFS concrete for both NAC and RAC mixtures at later ages is higher than that of the reference concrete without BFS. The rate of strength gains at ages of 56 and 91 days increases with the increase in BFS content of the BFS-based RAC. This trend of bond strength can be also observed in Figure 5 and Figure 6, in which the curve slope of the BFS mixtures at long-term ages tend to be steeper than that of the reference concrete without BFS. For instance, the strength gains in bond strength of the mixture RS00 at 28, 56 and 91 days with respect to 7-day strength of the same are 6%, 17% and 28%, respectively, obviously less than the RS45 mixture of 13%, 24% and 37%, and the RS75 mixture of 14%, 27% and 39%, respectively.

Based on these results, the BFS may provide the effects to enhance the long-term bond strength of RAC; the rate of strength gains at later ages beyond 56 days increase with the increase in BFS content. This may be due to the fact that the pozzolanic reaction of BFS can improve the strength properties of ITZ at the surface of steel bars, resulting in an enhancement of the long-term bond strength of the BFS concretes.

### 3.3. Durability Properties

#### 3.3.1. Resistance to Chloride-Ion Penetration

**Figure 7:**
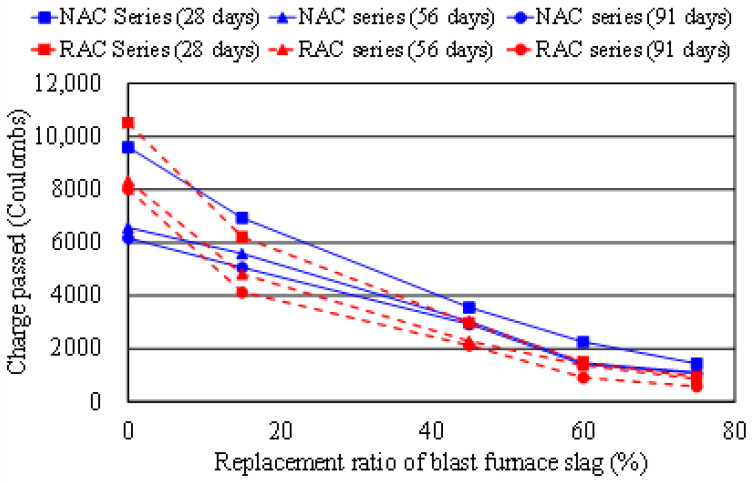
Total charge passed vs. BFS content of concrete.

Table 6 summarizes the measured results of the RCPT test for the resistance of concrete mixture to the chloride-ion penetration; the results are also shown in Figure 7. It is found that for most concrete mixtures of the NAC and RAC series, except the mixtures NS15 and RS15, the resistance to the chloride-ion penetration for each BFS concrete is significantly higher than that of the reference concrete, without BFS at later ages. When BFS is used, the chloride-ion penetration value of concrete decreases with an increase in BFS content (Figure 7) At 91 days, the total charge passed for mixture RS75 was 581 coulombs (C) (classified as very low permeability), far less when compared with 8114 C (high permeability) for the reference mixture RS00. Even at 28 days, the total charge passed for the mixture RS 75 was 938 C (very low permeability). This indicates that incorporating high volume (higher than 45%) of BFS in RAC at later ages may greatly reduce the chloride-ion ingress, and results in obvious improvement of the durability of concrete. This is believed to be due to the contribution of the pozzolanic character of BFS, which enhanced the resistance to chloride attack at long-term ages.

#### 3.3.2. Water Permeability

The water permeability (WP) of concrete was tested using a uniaxial flow apparatus performed on cylinder specimens (φ150 mm × 50 mm) subjected to 0.29 MPa pressure for 3 h. The WP was calculated with following formula:

(3) $$\mathrm{Water}\;\mathrm{permeability}=\frac{m_{2}-m_{1}}{m_{2}}\times 100\%$$

where *m*_1_ = initial weight of specimen and *m*_2_ = specimen weight after test.

Table 7 summarizes the measured WP of the concrete mixtures. It is seen that WP decreases with increasing compressive strength for both NAC and RAC mixtures. Incorporating BFS in concrete inherently reduces the WP of concrete at all ages. This is particularity found in mixtures NS 75 and RS 75 containing 75% BFS at curing age of 91 days, which has WP of 0.69% and 0.18%, respectively, comparably less with that of the reference mixture NS00 and RS00 of 0.46% and 0.79%, respectively. These results signify the fact that either NAC or RAC containing a high volume BFS at later ages may lead to lower water permeability, namely, superior durability to the concrete without BFS.

**Figure 8:**
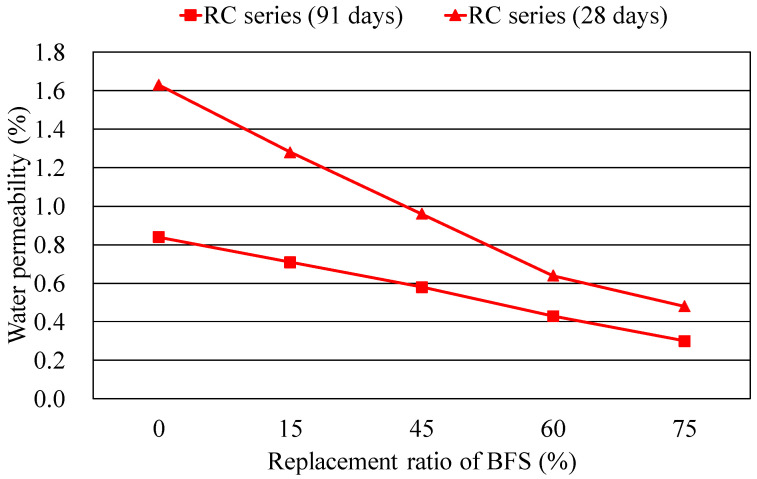
Influence of BFS content on the water permeability of RAC mixtures.

Furthermore, the effect on the water permeability of concrete by adding BFS can also be seen from Figure 8, which illustrates the relations between water permeability and BFS content of RAC mixture at curing ages of 28 and 91 days. Note that the WP of concrete decreases with the increase of BFS content for the two curing ages, while the curve trend for the 91-day concrete presents a steeper decline than that for the 28-day concrete, indicating that BFS concrete may exhibit less WP at later ages.

## 4. Conclusions

Based on the results and findings of the experimental work for concrete with w/cm ratio in the range of 0.30 to 0.50 and with BFS content in the range of 0% to 75% by weight of the total cementitious material as cement replacement, the following conclusions can be drawn:

1. The strength development with age of the NAC and RAC mixtures presents a similar trend, in which the compressive strength of RAC series is lower than that of the corresponding NAC series, indicating that, despite the cement replacing BFS, the mixtures of RAC result in reduced strength. In addition, the strength gain at later ages for the BFS mixtures is higher than that of the reference mixture without BFS.
2. Both of RAC and NAC mixtures incorporated with any BFS content show higher microhardness at ITZ than that of the correlated control mixture without BFS. Concrete at each age and containing more BFS presents higher microhardness at ITZ. This indicates that incorporating BFS makes the ITZ in concrete denser and stronger, especially at later ages.
3. The bond strength of BFS concretes is higher than that for the reference concrete without BFS, which increases with the increase in BFS content. The rate of bond strength gains at long-term ages with respect to the 7-day strength increases with the increase in BFS content. This may be due to pozzolanic reaction of BFS, which enhances the strength properties of ITZ at the surface of steel bars, resulting in an improvement of the long-term bond strength of BFS concretes.
4. Both RAC and NAC mixtures containing BFS present higher resistance to chloride ingress than that of the reference concrete without BFS, at all ages. The chloride-ion penetration value of the BFS-based concrete decreases with the increase in BFS content. Eventually, the mixture RS 75 comprising recycled aggregate and 75% BFS exhibits a pronounced resistance to chloride attack, revealing a chloride-ion penetration value of less than 1000 coulombs (very low permeability) at later ages.
5. Incorporating BFS in concrete can inherently reduce the water permeability of concrete at all ages. Either RAC or NAC containing high volume BFS (>45%) at later ages may lead to lower water permeability, namely, superior durability to the concrete with BFS.

## Figures and Tables

**Table 1:** Composition and physical properties of cement and blast furnace slag.

Components	Cement	Blast Furnace Slag
SiO_2_ (%)	21.4	38.1
Al_2_O_3_ (%)	4.9	7.5
Fe_2_O_3_ (%)	3.8	0.3
CaO (%)	64.2	39.9
MgO (%)	1.1	10.6
Na_2_O (%)	0.2	0.4
SO_3_ (%)	2.1	0.2
Loss on ignition (%)	2.1	1.4
Specific surface area (m^2^/kg)	336	538
Specific gravity	3.15	2.90

**Table 2:** Proportions of concrete mixtures.

Mixture	w/cm	Water	Cement	BFS ^b^	FA	NCA	RCA	SP
		kg/m^3^						
NS00 ^a^	0.50	210	420	0	800	860	0	1.1
NS15	0.46	193	357	63	839	860	0	1.0
NS30	0.42	176	294	126	878	860	0	1.8
NS45	0.38	160	231	189	918	860	0	2.6
NS60	0.34	143	168	252	956	860	0	3.6
NS75	0.30	126	105	315	995	860	0	6.3
RS00	0.50	210	420	0	793	0	750	1.1
RS15	0.46	193	357	63	832	0	750	1.5
RS30	0.42	176	294	126	871	0	750	2.3
RS45	0.38	160	231	189	910	0	750	3.6
RS60	0.34	143	168	252	950	0	750	5.9
RS75	0.30	126	105	315	989	0	750	8.7

Note: ^a^ N is normal aggregate concrete, R is recycled aggregate concrete; the number following the symbol S represents the percentage of cement replacement by BFS. ^b^ BFS: blast furnace slag, FA: fly ash, NCA: natural coarse aggregate concrete, RCA: recycled coarse aggregate, SP: superplasticizer.

**Table 3:** Properties of fresh concretes.

Mixture	Slump (mm)	Unit Weight (kg/m^3^)	Air Content (%)
NS00	225	2314	2.1
NS15	210	2303	1.2
NS30	220	2289	1.4
NS45	210	2285	2.3
NS60	250	2267	3.2
NS75	250	2236	3.4
RS00	215	2284	2.0
RS15	220	2273	1.8
RS30	250	2261	2.2
RS45	235	2238	3.1
RS60	230	2231	4.1
RS75	200	2215	4.8

**Table 4:** Compressive strengths of concretes (MPa).

Mixture	w/cm	Compressive Strength (MPa)			
		7 Days	28 Days	56 Days	91 Days
NS00	0.50	39.2 (100%)	42.1 (107%)	47.8 (122%)	56.3 (130%)
NS15	0.46	47.9 (100%)	55.4 (115%)	61.3 (128%)	64.2 (134%)
NS30	0.42	52.8 (100%)	62.9 (119%)	68.1 (129%)	71.3 (135%)
NS45	0.38	59.5 (100%)	65.6 (110%)	72.9 (123%)	78.8 (133%)
NS60	0.34	62.1 (100%)	72.3 (116%)	77.5 (125%)	82.4 (133%)
NS75	0.30	65.2 (100%)	76.2 (116%)	82.4 (126%)	86.2 (132%)
RS00	0.50	30.6 (100%)	32.6 (106%)	37.1 (121%)	38.3 (125%)
RS15	0.46	31.9 (100%)	37.1 (116%)	40.3 (126%)	44.6 (139%)
RS30	0.42	35.8 (100%)	40.3 (112%)	44.4 (124%)	49.7 (138%)
RS45	0.38	39.7 (100%)	46.7 (118%)	52.0 (130%)	54.6 (137%)
RS60	0.34	43.5 (100%)	51.1 (117%)	55.7 (128%)	58.5 (135%)
RS75	0.30	47.3 (100%)	53.6 (111%)	59.9 (127%)	64.1 (136%)

Note: The compressive strength was calculated as the average value from the test of 3 specimens for each type of mixture.

**Table 5:** Bond strength of concretes measured from pull-out tests.

Mixture	Age (day)	Concrete Strength (MPa)	Bond Strength *μ_avg_* (MPa)	Age (day)	Concrete Strength (MPa)	Bond Strength *μ_avg_* (MPa)
NS00	7	39.2	12.7 (100%)	56	47.8	15.1 (119%)
NS15		47.9	14.3 (100%)		61.2	17.2 (120%)
NS45		59.5	16.6 (100%)		72.9	20.5 (123%)
NS75		65.2	20.1(100%)		80.4	25.6 (127%)
RS00		30.6	9.8 (100%)		38.1	11.5 (117%)
RS15		31.9	10.1 (100%)		41.3	12.3 (122%)
RS45		39.7	11.9 (100%)		52.0	14.8 (124%)
RS75		47.3	13.2 (100%)		59.9	16.8 (127%)
NS00	28	42.1	13.9 (109%)	91	56.3	16.3 (128%)
NS15		55.4	16.0 (112%)		64.2	18.7 (131%)
NS45		65.6	18.8 (113%)		78.8	22.1 (133%)
NS75		76.2	23.4 (116%)		85.2	27.1 (135%)
RS00		32.6	10.4 (106%)		41.3	12.4 (126%)
RS15		37.1	11.3 (111%)		44.6	13.5 (134%)
RS45		46.2	13.5 (113%)		54.8	16.4 (138%)
RS75		52.6	15.0 (114%)		63.5	18.3 (139%)

**Table 6:** Measured results of the resistance to chloride-ion penetration of concretes.

Mixture	Total Charge Passed (Coulombs) *			Age (Days)
	28 Days	56 Days	91 Days	
NS00	9605	6567	6182	high/high/high **
NS15	6941	5589	5068	high/high/high
NS45	3562	3042	2928	moderate/moderate/moderate
NS60	2266	1478	1416	moderate/low/low
NS75	1446	1109	1076	low/low/low
RS00	10,506	8329	8014	high/high/high
RS15	6214	4813	4139	high/high/high
RS45	2973	2299	2136	moderate/moderate/moderate
RS60	1507	1385	922	low/low/very low
RS75	938	861	581	very low/very low/very low

* Average value of 3 specimens. ** Charge passed, chloride permeability, coulombs: >4000 C = high, 2000–4000 C = moderate, 1000–2000 C = low, 100–1000 C = very low and <100 C = negligible.

**Table 7:** Results of the concrete water permeability measurements.

Mixture	Compressive Strength (MPa)			Water Permeability (%)		
	28 Days	56 Days	91 Days	28 Days	56 Days	91 Days
NS00	42.1	47.8	51.3	0.52	0.47	0.46
NS15	55.4	61.3	64.2	0.46	0.41	0.40
NS45	65.6	72.9	78.8	0.36	0.28	0.21
NS60	72.3	77.5	82.4	0.25	0.22	0.16
NS75	76.2	82.4	86.2	0.15	0.13	0.09
RS00	32.6	39.1	38.3	0.84	0.81	0.79
RS15	37.1	40.3	44.6	0.71	0.68	0.57
RS45	46.7	52.0	54.6	0.58	0.49	0.38
RS60	51.1	55.7	58.5	0.43	0.32	0.21
RS75	52.6	59.9	64.1	0.30	0.23	0.18

## Data Availability

Data sharing is not applicable to this article.

## References

[1] Yaragal S.C., Teja D.C., Shaffi M. (2016). Performance studies on concrete with recycled coarse aggregates. Adv. Concr. Constr..

[2] Mao Y.A., Liu J.H., Shi C.J. (2021). Autogenous shrinkage and drying shrinkage of recycled aggregate concrete: A review. J. Clean. Prod..

[3] Nedeljković M., Visser J., Šavija B., Valcke S., Schlangen E. (2021). Use of fine recycled concrete aggregates in concrete: A critical review. J. Build. Eng..

[4] Yang K.H., Chung H.S., Ashour A.F. (2008). Influence of type and replacement level of recycled aggregates on concrete properties. ACI Mater. J..

[5] Limbachiya M.C., Leelawar T., Dhir R.K. (2000). Use of recycled concrete aggregate in high-strength concrete. Mater. Struct. Vol..

[6] Parekh D.N., Modhera C.D. (2011). Assessment of recycled aggregate concrete. J. Eng. Res. Stud..

[7] Gonza’lez-Fonteboa B., Martı´nez-Abella F., Eiras-Lo’pez J., Seara-Paz S. (2011). Effect of recycled coarse aggregate on damage of recycled concrete. Mater. Struct. Vol..

[8] (2018). ACI 232.2R, Report on the Use of Fly Ash in Concrete.

[9] (2005). ASTM C989, Standard Specification for Ground Granulated Blast-Furnace Slag for Use in Concrete and Mortars.

[10] (2009). CNS 12549, Ground Granulated Blast-Furnace Slag for Use in Concrete and Mortars.

[11] Ashish D.K., Singh B., Verma S.K. (2016). The effect of attack of chloride and sulphate on ground granulated blast furnace slag concrete. Adv. Concr. Constr..

[12] Kim D.J., Kim C.Y., Urgessac G., Choi J.H., Park C., Yeon J.H. (2019). Durability and rheological characteristics of high-volume ground-granulated blast-furnace slag concrete containing CaCO_3_/anhydrate-based alkali activator. Constr. Build. Mater..

[13] Duraman S.B., Richardson I.G. (2020). Microstructure & properties of steel-reinforced concrete incorporating Portland cement and ground granulated blast furnace slag hydrated at 20 °C. Cem. Concr. Res..

[14] Lee J.H., Lee T.Y. (2020). Durability and engineering performance evaluation of CaO content and ratio of binary blended concrete containing ground granulated blast-furnace slag. Appl. Sci..

[15] Nicula L.M., Corbu O., Iliescu M. (2020). Influence of blast furnace slag on the durability characteristic of road concrete such as freeze-thaw resistance. Procedia Manuf..

[16] Djelloul O.K., Menadi B., Wardeh G., Kenai S. (2018). Performance of self-compacting concrete made with coarse and fine recycled concrete aggregates and ground granulated blast-furnace slag. Adv. Concr. Constr..

[17] El-Hawary M., Al-Yaqout A., Nouh K. (2019). Durability of recycled aggregate concrete incorporating slag. Waste Resour. Manag..

[18] Khodair Y.A., Bommareddy B. (2017). Self-consolidating concrete using recycled concrete aggregate and high volume of fly ash, and slag. Constr. Build. Mater..

[19] Majhi R.K., Nayak A.N., Mukharjee B.B. (2018). Development of sustainable concrete using recycled coarse aggregate and ground granulated blast furnace slag. Constr. Build. Mater..

[20] Seara-Paz S., González-Fonteboa B., Eiras-López J., Herrador M.F. (2013). Bond behavior between steel reinforcement and recycled concrete. Mater. Struct..

[21] Butler L., West J.S., Tighe S.L. (2011). The effect of recycled concrete aggregate properties on the bond strength between RCA concrete and steel reinforcement. Cem. Concr. Res..

[22] Majhi R.K., Nayak A.N. (2019). Bond, durability and microstructural characteristics of ground granulated blast furnace slag based recycled aggregate concrete. Constr. Build. Mater..

[23] (2009). IS 10262, Concrete Mix Proportioning–Guidelines.

[24] Majhi R.K., Nayak A.N. (2020). Production of sustainable concrete utilising high-volume blast furnace slag and recycled aggregate with lime activator. J. Clean. Prod..

[25] Soylev T.A., François R. (2003). Quality of steel-concrete interface and corrosion of reinforcing steel. Cement and Concrete Research.

[26] Horne A.T., Richardson I.G., Brydson R.M.D. (2007). Quantitative analysis of the microstructure of interfaces in steel reinforced concrete. Cem. Concr. Res..

[27] Chen F.J., Li C.Q., Baji H., Ma B.G. (2018). Quantification of steel-concrete interface in reinforced concrete using Backscattered Electron imaging technique. Constr. Build. Mater..

[28] Igarashi S.B.A., Bentur A., Mindess S. (1996). Microhardness testing of cementation materials. Adv. Cem. Based Mater..

[29] Mindess S., Young J.F., Darwin D. (2003). Concrete.

[30] (1991). ACI 211.1, Standard Practice for Selecting Proportions for Normal, Heavyweight, and Mass Concrete.

[31] (2021). ASTM C39/C39M, Standard Test Method for Compressive Strength of Cylindrical Concrete Specimens.

[32] (1991). ASTM C234, Standard Test Method for Comparing Concretes on the Basis of the Bond Developed with Reinforcing Steel.

[33] (2012). ASTM C 1202, Standard Test Method for Electrical Indication of Concrete’s Ability to Resist Chloride Ion Penetration.

[34] (1965). IS 3085, Method of Test for Permeability of Cement Mortar and Concrete.

[35] (2017). ASTM E384, Standard Test Method for Microindentation Hardness of Materials.

